# Antimicrobial resistance and virulence in *Klebsiella pneumoniae*: a four-month study in Osogbo, Nigeria

**DOI:** 10.1017/ash.2025.21

**Published:** 2025-02-25

**Authors:** Taofikat Olatundun Akintoyese, Jude Oluwapelumi Alao, Elijah Kolawole Oladipo, Omolanke Temitope Oyedemi, Olubukola Monisola Oyawoye

**Affiliations:** 1 Microbiology Department, Adeleke University, Osun State, Nigeria; 2 School of Public Health and Interdisciplinary Studies, Auckland University of Technology, Auckland, New Zealand; 3 Department of Microbiology, Federal University, Oye-Ekiti, Ekiti State, Nigeria

## Abstract

**Objective:**

Antimicrobial resistance (AMR) is a growing global health crisis, with *Klebsiella pneumoniae* being a key pathogen due to its multidrug resistance (MDR). This study aimed to investigate the resistance profiles, demographic correlations, and molecular characteristics of MDR *K. pneumoniae* at UNIOSUN Teaching Hospital, Osogbo, Nigeria.

**Methods:**

From January to April 2022, 99 clinical isolates (*K. pneumoniae*) were collected from various specimen types (blood, sputum, urine, wound, stool, and oral cavity). Antibiotic susceptibility was assessed using the Kirby-Bauer disk diffusion method, and virulence genes were analysed using multiplex polymerase chain reaction.

**Results:**

All isolates exhibited resistance to ceftriaxone, cefotaxime, and colistin, with high resistance observed for cefepime and carbapenems (meropenem, imipenem, and ertapenem). Molecular characterization revealed the presence of virulence genes ***K1, K2,*** and ***mrkD*** in 15 isolates, while other tested virulence genes (***fimH, ramA, traT, K3,*** and ***K5***) were not detected. Significant associations were identified between resistance patterns and demographic factors, including age and sex, highlighting potential vulnerabilities in specific populations.

**Conclusions:**

This study underscores the alarming prevalence of MDR *K. pneumoniae* and aligns with global trends of rising AMR. Addressing these challenges requires targeted antimicrobial stewardship programs, infection control measures, public education, and enhanced surveillance systems. Incorporating molecular resistance testing and novel therapeutic agents in future research is crucial to developing effective containment strategies and preserving antibiotic efficacy.

## Introduction

Antimicrobial resistance (AMR) represents one of the greatest challenges to global public health in the 21st century, threatening to undermine decades of progress in treating bacterial infections.^1,2^ Among the most concerning pathogens is *Klebsiella pneumoniae* (*K. pneumoniae*), a member of the *Enterobacteriaceae* family that has emerged as a leading cause of multidrug-resistant (MDR) infections worldwide. The rapid evolution of resistance mechanisms in *K. pneumoniae* has significantly reduced the effectiveness of existing antibiotics, complicating treatment and leading to increased morbidity, mortality, and healthcare costs.^3^

*K. pneumoniae* is a common opportunistic pathogen associated with a wide range of infections, including respiratory tract infections, urinary tract infections (UTIs), bloodstream infections, liver abscesses, meningitis, and surgical wound infections.^4^ It is particularly problematic in hospital and community settings, where it ranks as the second most common opportunistic *Enterobacterium*, following *Escherichia coli*, and is a prominent member of the ESKAPE pathogens—a group of bacteria known for their ability to evade antibiotic treatments.^5^

The global rise of AMR in *K. pneumoniae* is particularly pronounced in low- and middle-income countries (LMICs), where the misuse and overuse of antibiotics, combined with inadequate antimicrobial stewardship programs, drive resistance development.^2,6^ WHO has classified carbapenem-resistant *K. pneumoniae* as a “critical priority pathogen,” underscoring the urgent need for research into its resistance mechanisms and novel therapeutic strategies.^7^ In LMICs, the burden of resistance is further exacerbated by the lack of access to advanced diagnostics and newer antibiotics,^8^ making it challenging to combat infections caused by carbapenem-resistant *Enterobacteriaceae* (CRE).

The resistance mechanisms in *K. pneumoniae* are diverse, involving the production of extended-spectrum β-lactamases (ESBLs), carbapenemases, and other enzymes that degrade antibiotics. Additionally, genetic adaptations such as efflux pumps, porin mutations, and horizontal transfer of resistance genes through plasmids contribute to its remarkable ability to resist multiple antibiotic classes.^12,13^ Carbapenems, often considered the last line of defence for treating severe infections caused by ESBL-producing bacteria, are increasingly ineffective due to rising resistance, leaving clinicians with limited treatment options.^9^

Beyond its resistance mechanisms, *K. pneumoniae* is equipped with a range of virulence factors, including capsular polysaccharides (eg, *K1* and K2 serotypes), adhesins, siderophores, and fimbriae, which enhance its ability to evade the host immune system and establish infections.^10^ Understanding the interplay between resistance and virulence is critical for designing effective therapeutic and preventive strategies.

Despite the global significance of *K. pneumoniae* as a multidrug-resistant pathogen, there is a limited understanding of its epidemiology and resistance mechanisms in LMICs, particularly in sub-Saharan Africa. Nigeria, for instance, faces significant challenges with AMR due to the widespread misuse of antibiotics, insufficient regulatory oversight, and limited availability of novel therapeutic agents.^11^ Surveillance efforts in these regions are often constrained by resource limitations, resulting in a lack of comprehensive data on resistance trends and molecular characteristics

This study addresses these gaps by investigating the AMR profiles, molecular characteristics, and virulence gene distribution of *K. pneumoniae* isolates from a tertiary healthcare facility in Nigeria. Specifically, we examine resistance to key antibiotic classes, carbapenem resistance prevalence, and virulence factors linked to capsular serotypes and pathogenicity. Additionally, we explore the associations between resistance patterns and demographic factors, providing valuable insights into the epidemiology of MDR *K. pneumoniae* in a resource-limited setting. By elucidating these critical aspects, the study aims to contribute to developing targeted antimicrobial therapies, effective stewardship programs, and containment strategies tailored to LMICs.

## Methods

### Ethical considerations

Ethical approval for this study was obtained from the Adeleke University Ethical Review Committee (AUERC) with reference number AUERC/FOS/MCB/10. The study adhered strictly to ethical guidelines for research involving human participants. Informed consent was obtained from all participants prior to specimen collection, and participants were assured of the confidentiality and anonymity of their personal and medical information. All data and specimens collected were securely stored and used solely for this research.

### Bacterial collection

UNIOSUN Teaching Hospital, formerly known as LAUTECH Teaching Hospital, is a prominent healthcare institution in Osogbo, Osun State, Southwest Nigeria, serving as a pivotal healthcare hub for the region. Between January and April 2022, a total of 200 clinical specimens were randomly collected from the hospital’s Microbiology department. These specimens included blood, sputum, urine, wound, stool, and oral cavity samples. All isolates were patient-unique, ensuring no duplicate samples from the same individual. The selection process was conducted randomly, with no attempts to balance isolates based on the various body sites of origin.

The samples were collected from patients at the study location using sterile swabs. The swab sticks were then placed in sterile coolers with icepacks maintained at 4–8°C upon collection and transported to the laboratory for analysis. Upon arrival at the laboratory, all samples were stored in freezers for long-term preservation until cultivation.

Patient information, including age, gender, admission class, diagnosis, and patient status, was obtained from medical records.

### Cultivation of samples and screening for enterobacteriaceae

Each sample was enriched in 225 mL of nutrient broth (Huankai Ltd., Guangzhou, China) and incubated at 37°C for 24 hours. After enrichment, the broth was streaked onto MacConkey agar (Huankai Ltd., Guangzhou, China) and incubated under the same conditions for an additional 24 hours. From the MacConkey agar plates, three pink mucoid colonies were selected and subcultured onto nutrient agar for 24 hours at 37°C.

The bacterial isolates were initially identified using Gram staining, capsule tests, and motility tests based on their morphological characteristics. Biochemical identification was conducted through a series of tests, including catalase, oxidase, citrate, urease, and indole tests. Sugar fermentation tests were performed using glucose, lactose, and sucrose, and Methyl Red/Voges-Proskauer (MR/VP) tests were also included. The identification of likely bacterial species was guided by *Bergey’s Manual of Systematic Bacteriology*. Confirmed cultures were preserved in Luria-Bertani broth supplemented with 20% glycerol and stored at −80°C for subsequent analyses.

### Antimicrobial sensitivity testing

AST was performed using the Kirby-Bauer disk diffusion method on Mueller-Hinton agar. This method was chosen for its simplicity, practicality, and standardization. A bacterial inoculum of approximately 1–2 × 10^8^ CFU/mL was evenly applied to the surface of Mueller-Hinton agar plates.

Six commercially prepared antibiotic disks with fixed concentrations were placed on the inoculated agar surface. The antibiotics tested were Ceftriaxone (5 μg), Cefotaxime (30 μg), Colistin (25 μg), Meropenem (10 μg), Imipenem (10 μg), and Ertapenem (10 μg). The plates were incubated at 35°C for 16–24 hours. After incubation, the growth inhibition zones around each antibiotic disk were measured in millimeters. The diameter of the inhibition zone was used to determine the isolates’ susceptibility, considering the antibiotics’ diffusion rate through the agar medium. The zone diameters of each drug were interpreted according to CLSI criteria.^12^ The classification of isolates as susceptible, intermediate (moderately resistant), or resistant was performed according to the Clinical and Laboratory Standards Institute (CLSI) breakpoints. *E. coli* ATCC 25922 was used as the quality control strain throughout the analysis.

### DNA extraction and multiplex PCR

DNA extraction was performed using a commercial kit from Stratec Biomedical Systems (Birkenfeld, Germany). A 1mL volume of bacterial suspension, standardized to .5 McFarland from an overnight culture, was centrifuged, and DNA was extracted from the resulting pellet according to the manufacturer’s instructions. The extracted DNA was resuspended in 100μL of TE buffer.

Serotyping primers (*K1, K2, K3*, and *K5*) and virulence-associated genes (*fimH, ramA, traT, mrkD*) were used for the identification and confirmation of *K. pneumoniae* strains. Primers for *K. pneumoniae* 16S–23S ITS were also used for strain confirmation, as described previously.^13^ The primers used in this study are shown in Table 1.

**Table 1.** Primers used for serotype and virulence gene identification

Gene	Primer Sequence (5′ → 3′)	Amplicon Size (bp)	Tm (°C)
***RmpA***	**Forward:** ACTGGGCTACCTCTGCTTCA	535	53
	**Reverse:** CTTGCATGAGCCATCTTTCA		
***fimH-1***	**Forward:** GCCAACGTCTACGTTAACCTG	180	43
	**Reverse:** ATATTTCACGGTGCCTGAAAA		
***mrkD***	**Forward:** CCACCAACTATTCCCTCGAA	226	43
	**Reverse:** ATGGAACCCACATCGACATT		
***traT***	**Forward:** GGTGTGGTGCGATGAGCACAG	288	55
	**Reverse:** CACGGTTCAGCCATCCCTGAG		
***K1***	**Forward:** GGTGCTCTTTACATCATTGC	1283	47
	**Reverse:** GCAATGGCCATTTGCGTTAG		
***K2***	**Forward:** GGATTATGACAGCCTCTCCT	908	45
	**Reverse:** CGACTTGGTCCCAACAGTTT		
***K5*** (Capsular Type)	**Forward:** TGGTAGTGATGCTCGCGA	280	49
	**Reverse:** CCTGAACCCACCCCAATC		
***K3***	**Forward:** TAGCAATTGACTTTAGGTG	549	—
	**Reverse:** AGTGAATCAGCCTTCACCT		

From the eventually used isolates, 20 isolates were randomly selected for serotyping and virulence gene testing. These isolates were chosen to represent diverse resistance patterns and clinical backgrounds, ensuring a representative sample for analysis. This selection allowed the study to focus on specific genetic traits linked to virulence and capsular types within a manageable sample size.

Two independent Multiplex PCR assays were performed to identify capsular serotypes and capsule-associated genes based on the protocol described previously.^14^ Each PCR was conducted in a 25 μL reaction mixture containing 5 pmol of each primer, 2 μL of extracted DNA, and the Master PCR mixture (Yekta Tajhiz Azma®, Iran). Amplified products were subjected to electrophoresis on a 1% agarose gel and stained with CyberSafe stain for visualization. The thermal cycling conditions for both Multiplex PCR assays were identical and followed established protocols.

### Statistical analysis

Statistical analyses were conducted using PyCharm (version 2024.1.2) with the scipy.stats library. The chi-square test was employed to examine associations between categorical variables, such as age group, sex, admission class, patient status, and sample type. Observed frequencies were compared to expected frequencies using the chi-square statistic, with a *p*-value of less than .05 considered statistically significant.

ANOVA was used to investigate differences in mean ages across categorical groups. Age was treated as a continuous variable, while sex, admission class, patient status, and sample type were considered categorical factors. Post hoc tests, including Tukey’s HSD, were conducted to determine specific group differences if ANOVA yielded significant results.

## Results

### Samples recruited for the study

Following selective isolation for *K. pneumoniae*, 101 samples were excluded due to the absence of growth on MacConkey agar, suggesting that these isolates were not part of the *Enterobacteriaceae* family. Consequently, 99 clinical samples that successfully grew on MacConkey agar were included in the study.

### Demographic and clinical characteristics

Table 2 summarizes the demographic and clinical characteristics of the study participants, including age and gender distribution, admission details, patient status, and sample site distribution.

**Table 2.** Demographic and clinical characteristics of study participants

Category	Subgroup	Frequency (n)	Percentage (%)
**Age Group**	16–20 years	8	8.08
	20–25 years	7	7.07
	26–30 years	21	21.21
	31–35 years	19	19.19
	36–40 years	22	22.22
	41–45 years	12	12.12
	46–50 years	3	3.03
	51–55 years	3	3.03
	56–60 years	3	3.03
	Unresponsive	1	1.01
**Gender**	Male	50	50.50
	Female	49	49.49
**Admission Ward**	Medical	73	73.73
	Surgical	26	26.26
**Patient Status**	In-patient	44	44.44
	Out-patient	55	55.55
**Sample Site**	Urine	18	18.18
	Blood	14	14.14
	Sputum	16	16.16
	Stool	15	15.15
	Oral cavity	18	18.18
	Wounds	18	18.18

### Bacterial characterization

All 99 isolates (100%) were identified as capsulated, Gram-negative bacilli, and were nonmotile. All isolates exhibited fermentation of glucose, lactose, and sucrose. They were uniformly positive for MR fermentation but negative for VP fermentation. Additionally, all isolates tested positive for citrate utilization, catalase activity, and urease production, but were negative for indole production and oxidase activity. Using *Bergey’s Manual of Systematic Bacteriology*, the isolates were conclusively identified as strains of *K. pneumoniae*.

### Antibiotic susceptibility

**Figure 1.**
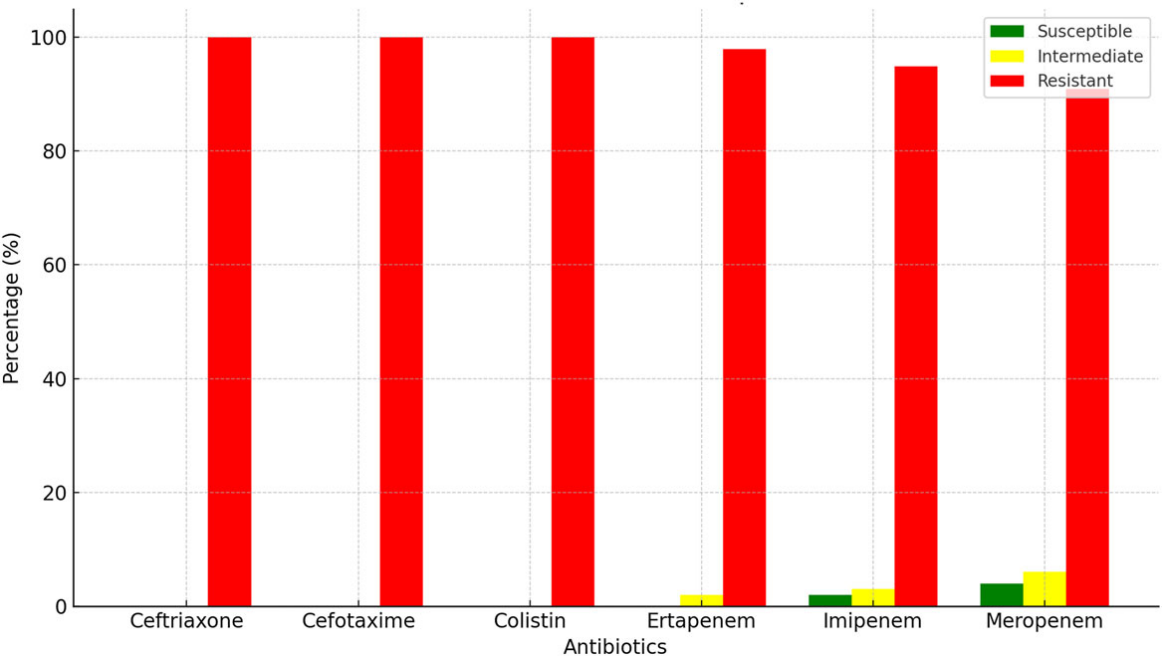
Antimicrobial resistance patterns of *K. pneumoniae* Isolates *Interpretation based on CLSI standard, 2020*.

The AMR patterns of *K. pneumoniae* isolates are summarized in Fig. 1. The figure illustrates the percentage of susceptible isolates that exhibited intermediate susceptibility or were resistant to each tested antibiotic. Notably, all isolates (100%) were resistant to ceftriaxone, cefotaxime, and colistin, while varying levels of resistance, intermediate susceptibility, and susceptibility were observed for ertapenem, imipenem, and meropenem.

### Serotype and virulence gene identification

Twenty clinical isolates were analyzed for serotype and virulence gene identification using Multiplex PCR. Among these, 15 isolates tested positive for the ***K1*** and ***K2*** serotypes. The ***mrkD*** virulence gene was also present in these 15 isolates.

In contrast, the remaining genes analyzed showed no amplification in the isolates. Specifically, ***fimH, ramA, traT, K3,*** and ***K5*** were negative across all isolates tested. These findings suggest that while the tested isolates predominantly carried the *K1* and *K2* serotypes and the mrkD virulence gene, other virulence and capsular-associated genes were absent in this sample set.

### Statistical analysis

The chi-square test revealed significant associations between demographic factors and antibiotic resistance patterns (*p*<.05). Specifically, age group and sex were significantly associated with resistance profiles. Admission class and patient status also showed significant associations. Additionally, significant associations were observed between sample type and sex.

ANOVA indicated a significant difference in mean ages across various categorical groups (*p*<.05). Post-hoc tests revealed specific group differences, suggesting that certain age groups had significantly different resistance patterns compared to others.

## Discussion

The emergence and dissemination of AMR in bacterial pathogens substantially threaten global public health. This study’s findings highlight the alarming prevalence of MDR *K. pneumoniae* strains in a clinical setting in Nigeria, consistent with global trends.^15,16^ By addressing the limited understanding of AMR in LMICs, particularly in Nigeria, this study provides real-world insights into resistance patterns and molecular characteristics of *K. pneumoniae*. The findings also demonstrate the critical role of demographic factors, resistance profiles, and virulence traits in shaping the epidemiology of AMR infections.

Our results revealed that all isolates exhibited resistance to ceftriaxone, cefotaxime, and cefepime. These findings are consistent with other studies in LMICs, where overuse and misuse of third-generation cephalosporins have led to high resistance rates.^17,18^ Furthermore, 35% of the isolates were resistant to carbapenems, often considered the last line of defence against severe gram-negative infections. This high level of carbapenem resistance underscores the global spread of CRE^19–21^ and aligns with the WHO’s prioritization of CRE as a critical public health threat.

The significant associations between demographic factors and resistance patterns provide further insights into the epidemiology of MDR *K. pneumoniae*. The chi-square test revealed that age and sex were significantly associated with resistance profiles, suggesting that specific demographic groups, such as males and older patients, are more vulnerable to MDR infections. These vulnerabilities may be attributed to increased healthcare exposure and comorbidities requiring frequent antibiotic use. Additionally, the analysis revealed significant differences in resistance patterns between isolates collected from different body sites, likely due to variations in local immune responses, antibiotic penetration, and site-specific prevalence of resistant strains. The high resistance observed in urinary and wound samples may reflect the selective pressure of antibiotics frequently used to treat these infections.

Molecular characterization of the isolates showed that all tested isolates harbored the virulence genes ***K1, K2***, and ***mrkD***, which are associated with capsule formation and adherence, enhancing the pathogen’s ability to evade host immune responses and persist in the host.^22,23^ These findings align with reports from similar studies, which have identified these virulence factors as critical contributors to the success of MDR *K. pneumoniae* in clinical settings.^24–26^ However, this study did not detect other tested genes, including ***fimH, ramA, traT, K3,*** and ***K5***, suggesting a potential variation in virulence profiles among isolates.

To provide additional clinical context, recent guidance from the Infectious Diseases Society of America (IDSA) highlights the use of novel therapeutic agents, such as meropenem-vaborbactam, ceftazidime-avibactam, imipenem-cilastatin-relebactam, and cefiderocol, for managing infections caused by AMR gram-negative bacteria, including *K. pneumonia*.^27^ These agents represent critical advancements in the treatment of CRE and other resistant infections. However, due to resource constraints and the limited availability of these agents in LMICs, this study did not include them in its resistance testing. Future studies should prioritize evaluating the efficacy of these agents in resource-limited settings to inform treatment guidelines better and improve clinical outcomes.

Comprehensive prevention strategies are essential to combat the spread of MDR *K. pneumoniae* and other AMR pathogens. Key strategies include antimicrobial stewardship programs (ASPs) to optimize antibiotic use, infection control measures such as strict hand hygiene and isolation precautions, and enhanced surveillance to track resistance patterns and detect outbreaks.^28^ Additionally, public education on the dangers of antibiotic misuse, regulated use of antibiotics in veterinary and agricultural practices, and investment in the research and development of new antibiotics and vaccines are critical. International collaboration and regulatory measures to control the sale and use of antibiotics are also needed to support these efforts.

This study has several limitations. AST was conducted exclusively using the disk diffusion method, which, although well-standardized and practical, provides limited quantitative insights compared to methods such as broth microdilution or automated systems. This study did not include molecular or genetic testing to identify specific resistance genes, such as common β-lactamase genes (eg, *CTX-M, AmpC*) or carbapenemase genes (eg, *NDM, OXA*). These genetic markers are critical for understanding resistance mechanisms and could provide valuable insights into the spread of resistance within *K. pneumoniae*. The absence of molecular characterization limits the ability to assess the full genetic basis of resistance and its clinical implications. Future studies should incorporate molecular resistance testing to provide a more comprehensive understanding of the genetic drivers of AMR in *K. pneumoniae.*

The study is further limited by its single-center design and relatively small sample size, which may restrict the generalizability of the findings. Additionally, the absence of testing for newer therapeutic agents recommended by the IDSA guidelines limits the clinical applicability of this study. Expanding testing capabilities to include novel agents and adopting molecular resistance analysis would enhance the depth and relevance of future research.

By addressing these challenges comprehensively, we can mitigate the impact of AMR and safeguard the efficacy of antibiotics for future generations. This study emphasizes the urgent need for targeted interventions, such as antimicrobial stewardship programs and the adoption of IDSA-recommended therapies, to improve clinical outcomes. Expanding testing capabilities and incorporating newer agents in LMICs is critical to developing effective strategies to combat MDR *K. pneumoniae* and other resistant pathogens.

## Conclusion

This study reveals a concerning prevalence of multidrug-resistant *K. pneumoniae* strains in a clinical setting in Nigeria. The findings highlight significant associations between demographic factors and resistance patterns, emphasizing the complexity of managing these infections. There is an urgent need for comprehensive antimicrobial stewardship programs, robust infection control measures, and continued research into novel therapeutic strategies and vaccines. Enhanced surveillance, public education, and international collaboration are essential to mitigate the spread of AMR. By addressing these challenges through targeted and coordinated efforts, we can preserve the effectiveness of antibiotics and protect public health.

## References

[1] Chinemerem Nwobodo D , Ugwu MC , Oliseloke Anie C , et al. Antibiotic resistance: the challenges and some emerging strategies for tackling a global menace. Clin Lab Anal 2022;36:e24655. 10.1002/jcla.24655 PMC9459344 35949048

[2] Salam MdA , Al-Amin MdY , Salam MT , et al. Antimicrobial resistance: a growing serious threat for global public health. Healthcare 2023;11:1946. 37444780 10.3390/healthcare11131946 PMC10340576

[3] Li Y , Kumar S , Zhang L , Wu H. Klebsiella pneumonia and its antibiotic resistance: a bibliometric analysis. BioMed Res Int 2022;2022:1–10. 10.1155/2022/1668789 PMC9192197 35707374

[4] Navon-Venezia S , Kondratyeva K , Carattoli A. Klebsiella pneumoniae: a major worldwide source and shuttle for antibiotic resistance. FEMS Microbiol Rev 2017;41:252–275. 28521338 10.1093/femsre/fux013

[5] Moya C , Maicas S. Antimicrobial resistance in Klebsiella pneumoniae strains: mechanisms and outbreaks. In: *The 1st International Electronic Conference on Microbiology*, MDPI; 2020: 11.

[6] Sharma A , Singh A , Dar MA , et al. Menace of antimicrobial resistance in LMICs: current surveillance practices and control measures to tackle hostility. J Infect Public Health 2022;15:172–181. 34972026 10.1016/j.jiph.2021.12.008

[7] Gürbüz M , Gencer G. Global trends and future directions on carbapenem-resistant Enterobacteriaceae (CRE) research: a comprehensive bibliometric analysis (2020–2024). Medicine 2024;103:e40783. 39654218 10.1097/MD.0000000000040783 PMC11630981

[8] Oliveira M , Antunes W , Mota S , Madureira-Carvalho Á , Dinis-Oliveira RJ , Dias Da Silva D. An overview of the recent advances in antimicrobial resistance. Microorganisms 2024;12:1920. 39338594 10.3390/microorganisms12091920 PMC11434382

[9] European Centre for Disease Prevention and Control. Carbapenem-Resistant Enterobacteriaceae, Second Update. Stockholm: ECDC; 2019.

[10] Schembri MA , Blom J , Krogfelt KA , Klemm P. Capsule and fimbria interaction in *Klebsiella pneumoniae* . Infect Immun 2005;73:4626–4633. 16040975 10.1128/IAI.73.8.4626-4633.2005 PMC1201234

[11] Iheanacho CO , Eze UIH. Antimicrobial resistance in Nigeria: challenges and charting the way forward. Eur J Hosp Pharm 2022;29:119. 35190457 10.1136/ejhpharm-2021-002762 PMC8899635

[12] CLSI. Performance Standards for Antimicrobial Suceptibility Testing, 30th edition. Wayne, PA, USA: Clinical and Laboratory Standards Institute; 2020.

[13] Turton JF , Perry C , Elgohari S , Hampton CV. PCR characterization and typing of Klebsiella pneumoniae using capsular type-specific, variable number tandem repeat and virulence gene targets. J Med Microbiol 2010;59:541–547. 20110386 10.1099/jmm.0.015198-0

[14] Candan ED , Aksöz N. Klebsiella pneumoniae: characteristics of carbapenem resistance and virulence factors. Acta Biochim Pol 2015;62:867–874. 26637376 10.18388/abp.2015_1148

[15] Afolayan AO , Oaikhena AO , Aboderin AO , et al. Clones and clusters of antimicrobial-resistant *Klebsiella* from Southwestern Nigeria. Clin Infect Dis. 2021;73(Suppl_4):S308–S315. doi:10.1093/cid/ciab769. 34850837 PMC8634535

[16] Akinyemi KO , Abegunrin RO , Iwalokun BA , et al. The emergence of Klebsiella pneumoniae with reduced susceptibility against third generation Cephalosporins and Carbapenems in Lagos Hospitals, Nigeria. Antibiotics 2021;10:142. 33535654 10.3390/antibiotics10020142 PMC7912815

[17] Moradigaravand D , Martin V , Peacock SJ , Parkhill J. Evolution and epidemiology of multidrug-resistant *Klebsiella pneumoniae* in the United Kingdom and Ireland. mBio 2017;8:e01976-16. 28223459 10.1128/mBio.01976-16 PMC5358916

[18] Mahmudul Hassan M. Scenario of antibiotic resistance in developing countries. In M Mare , S Hua Erin Lim , K-S Lai , R-T Cristina , editors. Antimicrobial Resistance – A One Health Perspective. IntechOpen; 2021.

[19] Pham MH , Hoi LT , Beale MA , et al. Evidence of widespread endemic populations of highly multidrug resistant Klebsiella pneumoniae in hospital settings in Hanoi, Vietnam: a prospective cohort study. Lancet Microbe 2023;4:e255–e263. 36801013 10.1016/S2666-5247(22)00338-X

[20] Satlin MJ , Jenkins SG , Walsh TJ. The global challenge of Carbapenem-resistant Enterobacteriaceae in transplant recipients and patients with hematologic malignancies. Clin Infect Dis 2014;58:1274–1283. 24463280 10.1093/cid/ciu052 PMC4038783

[21] Wise MG , Karlowsky JA , Mohamed N , et al. Global trends in carbapenem- and difficult-to-treat-resistance among World Health Organization priority bacterial pathogens: ATLAS surveillance program 2018–2022. J Global Antimicrob Resist 2024;37:168–175. 10.1016/j.jgar.2024.03.020 38608936

[22] Kot B , Piechota M , Szweda P , et al. Virulence analysis and antibiotic resistance of Klebsiella pneumoniae isolates from hospitalised patients in Poland. Sci Rep 2023;13:4448. 36932105 10.1038/s41598-023-31086-w PMC10023695

[23] Compain F , Babosan A , Brisse S , et al. Multiplex PCR for detection of seven virulence factors and K1/K2 Capsular Serotypes of Klebsiella pneumoniae. J Clin Microbiol 2014;52:4377–4380. 25275000 10.1128/JCM.02316-14 PMC4313302

[24] Effah CY , Sun T , Liu S , Wu Y. Klebsiella pneumoniae: an increasing threat to public health. Ann Clin Microbiol Antimicrob 2020;19:1. 31918737 10.1186/s12941-019-0343-8 PMC7050612

[25] Bakhtiari R , Javadi A , Aminzadeh M , Molaee-Aghaee E , Shaffaghat Z. Association between presence of RmpA, MrkA and MrkD genes and antibiotic resistance in clinical Klebsiella pneumoniae isolates from hospitals in Tehran, Iran. *IJPH* 2021. 10.18502/ijph.v50i5.6118 PMC8223560 34183959

[26] Ghasemian A , Mobarez AM , Peerayeh SN , Bezmin Abadi AT. The association of surface adhesin genes and the biofilm formation among Klebsiella oxytoca clinical isolates. New Microbes New Infect 2019;27:36–39. 30581573 10.1016/j.nmni.2018.07.001 PMC6290254

[27] Tamma PD , Heil EL , Justo JA , Mathers AJ , Satlin MJ , Bonomo RA. Infectious diseases society of America 2024 guidance on the treatment of antimicrobial-resistant Gram-negative infections. *Clin Infect Dis* 2024:ciae403. 10.1093/cid/ciae403 39108079

[28] Assi M , Abbas S , Nori P , et al. Infection prevention and antimicrobial stewardship program collaboration during the COVID-19 pandemic: a window of opportunity. Curr Infect Dis Rep 2021;23:15. 34426728 10.1007/s11908-021-00759-w PMC8374122

